# Psychiatric Morbidity, Caregiver Burden, and Quality of Life Among Caregivers of Patients With Cancer: A Cross-Sectional Study From a Tertiary Care Center in India

**DOI:** 10.7759/cureus.107350

**Published:** 2026-04-19

**Authors:** Kosha Thorat, Mahesh Suthar, Aditya Mhaskar, Vidhi Modi

**Affiliations:** 1 Psychiatry, Baroda Medical College and SSG (Sir Sayajirao General) Hospital, Vadodara, IND; 2 Psychiatry, GMERS (Gujarat Medical Education and Research Society) Medical College and Hospital Valsad, Valsad, IND; 3 Psychiatry, Healthy Mind Hospital, Navrangpura, Ahmedabad, IND

**Keywords:** anxiety, cancer caregivers, caregiver burden, caregiver quality of life, depression, psycho-oncology

## Abstract

Background: Cancer is a major cause of morbidity and mortality worldwide and has a profound psychological impact not only on patients but also on their caregivers. Caregivers often experience significant emotional distress, including anxiety, depression, and caregiver burden, which may adversely affect their quality of life.

Methods: A hospital-based cross-sectional study was conducted over six months (January 2021 to June 2021) at the Radiation Oncology Department of SSG Hospital, Vadodara, India. A total of 400 caregivers of cancer patients were recruited. Data were collected using a structured proforma. Caregiver burden was assessed using the Zarit Burden Interview, and quality of life was assessed using the Caregiver Quality of Life Index-Cancer (CQOLC). Psychiatric morbidity was assessed through clinical interviews, and diagnoses of depression and anxiety were made based on DSM-5 (Diagnostic and Statistical Manual of Mental Disorders, 5th Edition) criteria. Severity was further evaluated using the Hamilton Depression Rating Scale and Hamilton Anxiety Rating Scale.

Results: Among 400 caregivers, 42% were diagnosed with depression and 18% with anxiety based on clinical evaluation. A substantial proportion of caregivers experienced caregiver burden, with 40% reporting mild to severe levels. The mean caregiver burden score was 19.18 ± 12.28, and the mean quality-of-life score was 98.03 ± 12.46, indicating moderate impairment. Caregiver burden was significantly associated with sociodemographic variables and illness-related factors, including advanced stage of cancer.

Conclusion: Caregivers of cancer patients experience substantial psychological distress, caregiver burden, and reduced quality of life. These findings highlight the need for routine psychological screening and structured psychosocial interventions targeting caregivers as an integral component of comprehensive cancer care.

## Introduction

Cancer remains a major global health concern and is one of the leading causes of morbidity and mortality worldwide. In India, the burden of cancer is steadily increasing, posing significant challenges not only to patients but also to their families and caregivers. Advances in early diagnosis and treatment have improved survival rates; however, these improvements have shifted a substantial portion of caregiving responsibilities to family members, particularly in low- and middle-income countries where formal support systems are limited [1,2].

Caregivers play a pivotal role in the management of cancer patients by providing physical care, emotional support, and financial assistance throughout the illness trajectory. While this role is essential, it is often associated with considerable psychological and physical strain. Caregivers frequently experience anxiety, depression, emotional exhaustion, and deterioration in quality of life (QOL) due to prolonged caregiving demands, financial constraints, and uncertainty regarding disease prognosis [3].

The concept of caregiver burden encompasses the multidimensional stress experienced while caring for a chronically ill individual, including emotional, social, financial, and physical domains. Standardized instruments such as the Zarit Burden Interview (ZBI) and the Caregiver Quality of Life Index-Cancer (CQOLC) have been widely used to objectively assess caregiver burden and QOL in oncology settings [4,5].

Despite increasing global recognition of caregiver distress, there remains a relative paucity of comprehensive research in the Indian context evaluating caregiver burden, psychiatric morbidity, and QOL simultaneously. Most existing studies focus on isolated aspects, thereby limiting a holistic understanding of caregiver challenges [2].

Given this gap in the literature, the present study aims to assess caregiver burden, QOL, and the prevalence of depression and anxiety among caregivers of cancer patients, along with their associated sociodemographic and illness-related factors. This study seeks to provide clinically relevant insights that may contribute to the development of integrated, caregiver-focused interventions in oncology care

## Materials and methods

Study design and setting

This was a hospital-based cross-sectional study conducted at the Radiation Oncology Department of SSG Hospital, Vadodara, Gujarat, India.

Study period

The study was conducted over six months from January 2021 to June 2021.

Participants

A total of 400 caregivers of cancer patients were recruited. A caregiver was defined as an individual primarily responsible for the patient’s care for at least six months.

Inclusion and exclusion criteria

Caregivers aged 18 years and above who were primary caregivers of cancer patients for a duration of at least six months and who provided informed consent were included in the study. Caregivers with a known history of psychiatric illness prior to the caregiving period and those unwilling to participate were excluded from the study.

Data collection

Data were collected through face-to-face interviews using a self-developed semi-structured proforma.

Study instruments

The Zarit Burden Interview (ZBI-22) was used to assess caregiver burden. It is a validated 22-item Likert scale widely used to assess caregiver burden across multiple domains [4]. The CQOLC scale was used to assess QOL. It is a cancer-specific, validated instrument that evaluates multiple domains of caregiver well-being [5].

Psychiatric morbidity was assessed through detailed clinical interviews, and diagnoses were made according to the Diagnostic and Statistical Manual of Mental Disorders-5 (DSM-5), which provides standardized diagnostic guidelines for mental disorders [6]. Severity of depression and anxiety was further assessed using the Hamilton Depression Rating Scale (HAM-D) and the Hamilton Anxiety Rating Scale (HAM-A) [7,8].

The Zarit Burden Interview (ZBI) and Hamilton rating scales are available for academic and research use with appropriate citation. The DSM-5 criteria were used strictly for diagnostic purposes and were not reproduced in the manuscript. The CQOLC is a standardized instrument used in research settings and was applied in accordance with its published guidelines, with appropriate citations. 

Psychiatric assessments were conducted through detailed clinical interviews by psychiatry residents under the supervision of consultant psychiatrists (MD Psychiatry) with clinical experience in diagnostic evaluations. Interviews were conducted in a standardized manner according to DSM-5 diagnostic criteria. Each interview included a comprehensive psychiatric history, mental status examination, and relevant psychosocial assessment.

Wherever feasible, collateral history was obtained from accompanying family members to improve diagnostic accuracy. The average duration of each assessment ranged from 20 to 30 minutes.

Although no structured diagnostic interview was used, efforts were made to ensure consistency by adhering to DSM-5 criteria and by supervision from senior psychiatrists.

To maintain diagnostic reliability, cases with diagnostic uncertainty were reviewed and discussed with a senior consultant psychiatrist before final diagnosis

Sample size calculation

The sample size was calculated using the standard formula for estimating proportions:

$n = \frac{Z^{2}\times p \times (1 &minus; p)}{d^{2}}$

where Z is the standard normal deviate at 95% confidence level (1.96), p is the expected prevalence (assumed to be 50% due to lack of prior data), and d is the allowable error (5%). The calculated sample size was approximately 384; however, 400 participants were included to ensure better representation.

Statistical analysis

Data were analyzed using MedCalc Statistical Software version 20.0 (MedCalC Software Ltd, Ostend, Belgium). Descriptive statistics were used. Descriptive statistics were used to summarize sociodemographic and clinical characteristics (Table 1, Table 2). The Chi-square test was used to assess the association between categorical variables, such as sociodemographic factors and psychiatric outcomes (Tables 4, 5). Independent t-tests and ANOVA were used to compare mean scores for caregiver burden and QOL across groups. A p-value of less than 0.05 was considered statistically significant.

All analyses were conducted following standard epidemiological reporting guidelines for cross-sectional studies.

Ethical approval

Approved by the Institutional Ethics Committee of Medical College and SSG Hospital, Vadodara (Approval No: IECBHR/85-2021).

## Results

A total of 400 caregivers were included in the study. The majority were aged less than 45 years (58%), with males constituting 57% of the sample. Most participants were married (84.2%) and belonged to nuclear families (63.3%). Nearly half had secondary education (47.7%), and 22% were unemployed. More than half (56.5%) belonged to a lower socioeconomic status. The majority of caregivers were first-degree relatives (65.8%), followed by spouses (23%) and second-degree relatives (11.3%) (Table 1).

**Table 1:** Sociodemographic characteristics of caregivers (N = 400)

Variable	Category	n (%)
Age	<45 years	232 (58.0)
	≥45 years	168 (42.0)
Sex	Male	228 (57.0)
	Female	172 (43.0)
Religion	Hindu	373 (93.3)
	Muslim	27 (6.7)
Residence	Rural	188 (47.0)
	Urban	212 (53.0)
Education	Illiterate	50 (12.5)
	Primary	68 (17.0)
	Secondary	191 (47.7)
	Graduate+	91 (22.8)
Occupation	Unemployed	88 (22.0)
	Unskilled	175 (43.7)
	Skilled	137 (34.2)
Income	Lower	226 (56.5)
	Lower-middle	97 (24.3)
	Middle	61 (15.3)
	Upper-middle	16 (4.0)
Marital status	Married	337 (84.2)
	Others	63 (15.8)
Family type	Joint	147 (36.7)
	Nuclear	253 (63.3)
Relationship	Spouse	92 (23.0)
	First-degree	263 (65.8)
	Second-degree	45 (11.3)

The illness-related characteristics of the patients are shown in Table 2. Among the patients, breast and cervix cancers (38.5%) were the most common, followed by oral cancers (31.2%). A large proportion of patients were in advanced stages, with Stage III (33.5%) and Stage IV (43.3%) constituting the majority. Most patients were receiving chemotherapy (55.2%), and 78.3% had a treatment duration of less than two years.

**Table 2:** Illness-related characteristics of patients (N = 400)

Variable	Category	n (%)
Type of cancer	Oral	125 (31.2)
	Breast/cervix	154 (38.5)
	Neck/thorax	81 (20.3)
	Abdominal	40 (10.0)
Stage	Stage I	14 (3.5)
	Stage II	79 (19.8)
	Stage III	134 (33.5)
	Stage IV	173 (43.3)
Treatment	Chemotherapy	221 (55.2)
	Radiotherapy	17 (4.2)
	CT + RT	131 (32.7)
	Brachytherapy	31 (7.7)
Duration	<2 years	313 (78.3)
	≥2 years	87 (21.8)

Caregiver burden distribution is presented in Table 3, with the majority experiencing minimal to moderate burden. Domain-wise burden and quality-of-life (QOL) scores are summarized in Table 4. 

Assessment of caregiver burden revealed that the majority (59.7%) experienced minimal or no burden. However, 33.5% had mild to moderate burden, and 6.7% reported moderate to severe burden. The mean burden score was 19.18 ± 12.28, indicating a mild overall burden (Table 3).

**Table 3:** Distribution of caregiver burden (ZBI) ZBI: Zarit Burden Interview. Chi-square test was used for categorical variables. Independent t-test and ANOVA were used for continuous variables.

Burden level	n (%)
No/minimal	239 (59.7)
Mild–moderate	134 (33.5)
Moderate–severe	27 (6.7)

Domain-wise analysis showed that emotional strain and relationship-related burden contributed more significantly compared to financial burden (Table 4).

**Table 4:** Mean scores of burden and quality of life domains SD: standard deviation, QOL: quality of life. Chi-square test was used for categorical variables. Independent t-test and ANOVA were used for continuous variables.

Domain	Mean	SD
Total burden	19.18	12.28
Emotional well-being	5.94	4.17
Social/family life	3.44	2.38
Financial burden	1.15	0.38
Loss of control	3.47	2.38
Total QOL	98.03	12.46

The mean total QOL score was 98.03 ± 12.46, suggesting moderately preserved QOL among caregivers. Higher scores were observed in domains related to disruptiveness and burden adaptation, whereas lower scores were noted in financial concerns and positive adaptation domains.

Based on clinical diagnostic assessment, depression was present in 42% of caregivers, and anxiety disorders were present in 18% caregivers. Among those with depression, the majority had mild severity, followed by moderate and severe forms. Similarly, most caregivers with anxiety had mild to moderate symptoms, with fewer having severe anxiety.

The association between sociodemographic variables and depression and anxiety is shown in Table 5.

**Table 5:** Association of sociodemographic variables with depression and anxiety Chi-square test was used for categorical variables. Independent t-test and ANOVA were used for continuous variables.

Variable	Depression (p-value)	Anxiety (p-value)
Sex	0.0026	0.0254
Residence	0.0252	<0.0001
Education	0.4668	0.0002
Occupation	0.1410	0.0079
Income	0.2617	0.0015

Significant associations were observed between psychiatric morbidity and various sociodemographic factors. Depression was significantly higher among females, rural residents, and first-degree relatives. Anxiety was significantly associated with female gender, rural residence, lower education levels, unemployment, lower income, and substance use. Caregiver burden was significantly higher among older caregivers (>45 years), those with lower education, unemployed individuals, and lower income groups. QOL was significantly better among younger caregivers, males, urban residents, and those with higher education and income.

Illness-related factors also showed significant associations with caregiver outcomes. Advanced cancer stage (Stage III and IV) was significantly associated with higher caregiver burden, lower QOL, and increased depression. Shorter duration of treatment (<2 years) was associated with a higher prevalence of depression. Type of cancer showed a significant association with QOL and anxiety, with relatively better QOL observed in caregivers of breast and cervical cancer patients.

Caregiver outcomes varied depending on their relationship with the patient. Higher burden scores were observed among spouses and first-degree relatives. Depression was more prevalent among first-degree relatives. Anxiety was more common among spousal caregivers.

Caregivers with depression had significantly higher burden scores compared to non-depressed caregivers (p = 0.004). Both depression and anxiety were associated with significantly lower QOL scores, indicating a strong relationship between psychological morbidity and caregiver well-being.

## Discussion

In this study, psychiatric morbidity was assessed using clinical diagnostic criteria rather than screening tools. A substantial proportion of caregivers of cancer patients were found to experience psychiatric morbidity, with 42% diagnosed with depression and 18% with anxiety, highlighting the significant psychological burden associated with caregiving. These findings are consistent with previous literature, where caregiver distress ranges between 30% and 60% globally [9,10]. Caregivers often experience emotional strain due to uncertainty regarding prognosis, financial stress, and prolonged caregiving responsibilities [3].

The mean burden score in this study (19.18) indicates predominantly mild burden, similar to findings by Lukhmana et al., who reported a comparable burden profile among caregivers in India [11]. However, lower burden scores than those reported in studies such as Mishra et al. may reflect differences in patient severity and healthcare settings [12]. Higher burden among unemployed and low-income caregivers highlights the role of financial instability in exacerbating caregiver stress, consistent with previous findings [13]. Furthermore, the association between advanced cancer stage and increased caregiver burden is clinically expected, as later stages involve greater symptom burden, dependency, and emotional distress [14].

The prevalence of depression (42%) in this study is comparable to findings by Rahmani et al. [15], though lower than some Indian studies reporting higher prevalence [16]. Such variability may be attributed to differences in study design, population characteristics, and assessment methods. Female caregivers showed significantly higher rates of depression, consistent with established literature suggesting greater emotional vulnerability and caregiving involvement among women [17]. A higher prevalence among first-degree relatives and spouses likely reflects greater emotional involvement and caregiving responsibilities, as reported in previous studies [18].

The prevalence of anxiety (18%) aligns with findings from similar studies [19]. Anxiety was more common among females, rural residents, and individuals with lower levels of education. Caregivers from rural backgrounds may experience higher levels of anxiety due to limited access to healthcare services, financial constraints, and inadequate social support systems, particularly in low-resource settings.

The mean QOL score (98.03) indicates moderate impairment, consistent with prior studies [20]. QOL was significantly influenced by socioeconomic factors, including education, income, and occupation. Urban caregivers demonstrated better QOL, likely due to improved access to healthcare facilities and support services, as well as greater financial stability. These findings are consistent with previous Indian and international studies [21]. Additionally, caregivers of patients with early-stage cancer reported better QOL, underscoring the impact of disease severity on caregiver well-being.

Depression was significantly associated with increased caregiver burden and reduced QOL, suggesting a bidirectional relationship. Psychological distress may impair coping ability, thereby increasing perceived burden. Similarly, anxiety was associated with poorer QOL, indicating that emotional distress directly affects caregiver functioning. These findings emphasize the need for integrated psychosocial interventions targeting caregivers.

Clinical implications

The findings of this study highlight the critical need to integrate caregiver-focused mental health services into routine oncology care. A substantial proportion of caregivers experience depression, anxiety, and varying levels of burden, which can adversely affect not only their own well-being but also the quality of care provided to patients. Early identification through regular psychological screening, including clinical interviews and brief, validated tools, can help detect at-risk caregivers. Particular attention should be given to high-risk groups identified in this study, including female caregivers, individuals from rural backgrounds, those with lower socioeconomic status, and caregivers of patients with advanced-stage cancer. Incorporating structured psychoeducation, stress management strategies, and supportive counseling into oncology settings can significantly reduce caregiver distress. Additionally, strengthening social support systems, facilitating access to financial and community resources, and promoting caregiver support groups can improve coping and resilience. Training healthcare professionals to recognize caregiver strain and encouraging a multidisciplinary approach involving psychiatrists, psychologists, and oncologists can further enhance outcomes. Ultimately, addressing caregiver mental health is essential for holistic cancer care, as improved caregiver well-being directly contributes to better patient adherence, recovery, and overall treatment outcomes.

Limitations

This study was conducted in a single tertiary care center and excluded caregivers of patients undergoing surgical treatment, which may limit generalizability. The cross-sectional design restricts causal inference. Multicentric longitudinal studies are recommended to further validate these findings. Additionally, the absence of a standardized screening tool due to licensing constraints is a limitation; however, diagnosis based on clinical criteria enhances real-world applicability.

## Conclusions

Caregivers of cancer patients experience substantial psychological burden, with a significant proportion exhibiting symptoms of depression and anxiety. In addition to emotional distress, caregivers also face considerable challenges in terms of reduced QOL, particularly those belonging to lower socioeconomic strata, rural backgrounds, and those caring for patients with advanced stages of cancer. The findings of this study highlight that both sociodemographic factors (such as age, gender, education, and income) and illness-related factors (such as cancer stage and treatment duration) play a crucial role in determining caregiver burden and psychological well-being. The strong association observed between caregiver burden, psychiatric morbidity, and reduced QOL underscores the need for early identification of at-risk caregivers.

Given the central role caregivers play in the continuum of cancer care, their mental health and well-being should be considered an integral component of comprehensive oncology management. Incorporating routine psychological screening, providing psychoeducation, and offering structured psychosocial interventions for caregivers may not only improve their QOL but also positively influence patient outcomes and treatment adherence. Future research should focus on longitudinal, multicenter studies to better understand the evolving needs of caregivers and develop targeted, culturally appropriate intervention strategies.
